# Finite-size correction scheme for supercell calculations in Dirac-point two-dimensional materials

**DOI:** 10.1038/s41598-018-27632-6

**Published:** 2018-06-19

**Authors:** C. G. Rocha, A. R. Rocha, P. Venezuela, J. H. Garcia, M. S. Ferreira

**Affiliations:** 1 School of Physics, Trinity College Dublin, Dublin 2, Ireland; 2 Centre for Research on Adaptive Nanostructures and Nanodevices (CRANN), Trinity College Dublin, Dublin 2, Ireland; 3 Advanced Materials and Bioengineering Research Centre (AMBER), Trinity College Dublin, Dublin 2, Ireland; 4 Instituto de Física Teórica, Universidade Estadual Paulista (Unesp), São Paulo, Brazil; 5 Department of Chemical Engineering, Massachusetts Institute Of Technology, Cambridge, MA 02139 USA; 6 Instituto de Física, Universidade Federal Fluminense, Niterói, RJ Brazil; 7 Catalan Institute of Nanoscience and Nanotechnology (ICN2), Barcelona, Spain; 8 CSIC The Barcelona Institute of Science and Technology, Campus UAB, Bellaterra, 08193 Barcelona, Spain

## Abstract

Modern electronic structure calculations are predominantly implemented within the super cell representation in which unit cells are periodically arranged in space. Even in the case of non-crystalline materials, defect-embedded unit cells are commonly used to describe doped structures. However, this type of computation becomes prohibitively demanding when convergence rates are sufficiently slow and may require calculations with very large unit cells. Here we show that a hitherto unexplored feature displayed by several 2D materials may be used to achieve convergence in formation- and adsorption-energy calculations with relatively small unit-cell sizes. The generality of our method is illustrated with Density Functional Theory calculations for different 2D hosts doped with different impurities, all of which providing accuracy levels that would otherwise require enormously large unit cells. This approach provides an efficient route to calculating the physical properties of 2D systems in general but is particularly suitable for Dirac-point materials doped with impurities that break their sublattice symmetry.

## Introduction

Modern tools to calculate physical and chemical properties of materials have reached unprecedented accuracy levels^1^. Most of these tools are based on *ab initio* atomistic calculations of the electronic structure of materials often described within small unit cells that are periodically repeated to span the entire space. The use of such periodic boundary conditions (PBC) is the norm not only in the treatment of pristine crystalline materials. In fact, supercell calculations consisting of identical defect-embedded cells periodically arranged in space are also the standard for studying non-crystalline structures and are believed to capture the essential characteristics of disordered materials containing dopants^2^.

While the PBC approach permits the use of well-established computational packages available for materials properties simulations^3–5^ based on Density Functional Theory (DFT)^6,7^, cell sizes must be sufficiently large to avoid the interaction between the periodically spaced defects. Problems are known to arise when convergence is very slow and reached only with cell sizes that make the calculations too demanding. Several attempts have been made to introduce finite-size supercell corrections in order to mitigate the computational costs of such calculations but they are primarily aimed at 3D materials and not necessarily at their 2D counterparts^8–14^.

One peculiar feature of several 2D materials that may aggravate the slow-convergence problem even further is when impurity-induced Friedel oscillations become perfectly commensurate with the underlying lattice. In this case, the interaction between impurities that would otherwise vanish is enhanced and may retard the convergence of supercell calculations quite considerably^15^. However, rather than accepting this feature as a hindrance, here we explore this peculiarity seen in many 2D materials to introduce a finite-size supercell correction scheme that reduces the computational costs and/or increases the accuracy levels of calculations in these materials by orders of magnitude. The goal of our formulation is to determine energy variation quantities associated to a host material being perturbed by the presence of an impurity. This is done via an analytic approach that gives the energetics of the perturbed system in a transparent functional form. This work is a generalization of a correction scheme we developed originally for carbon nanotubes^14^. Although carbon nanotubes are quasi-one-dimensional structures, they share some key characteristics of 2D materials as they can be thought of as rolled up graphene sheets.

## Methods

**Figure 1:**
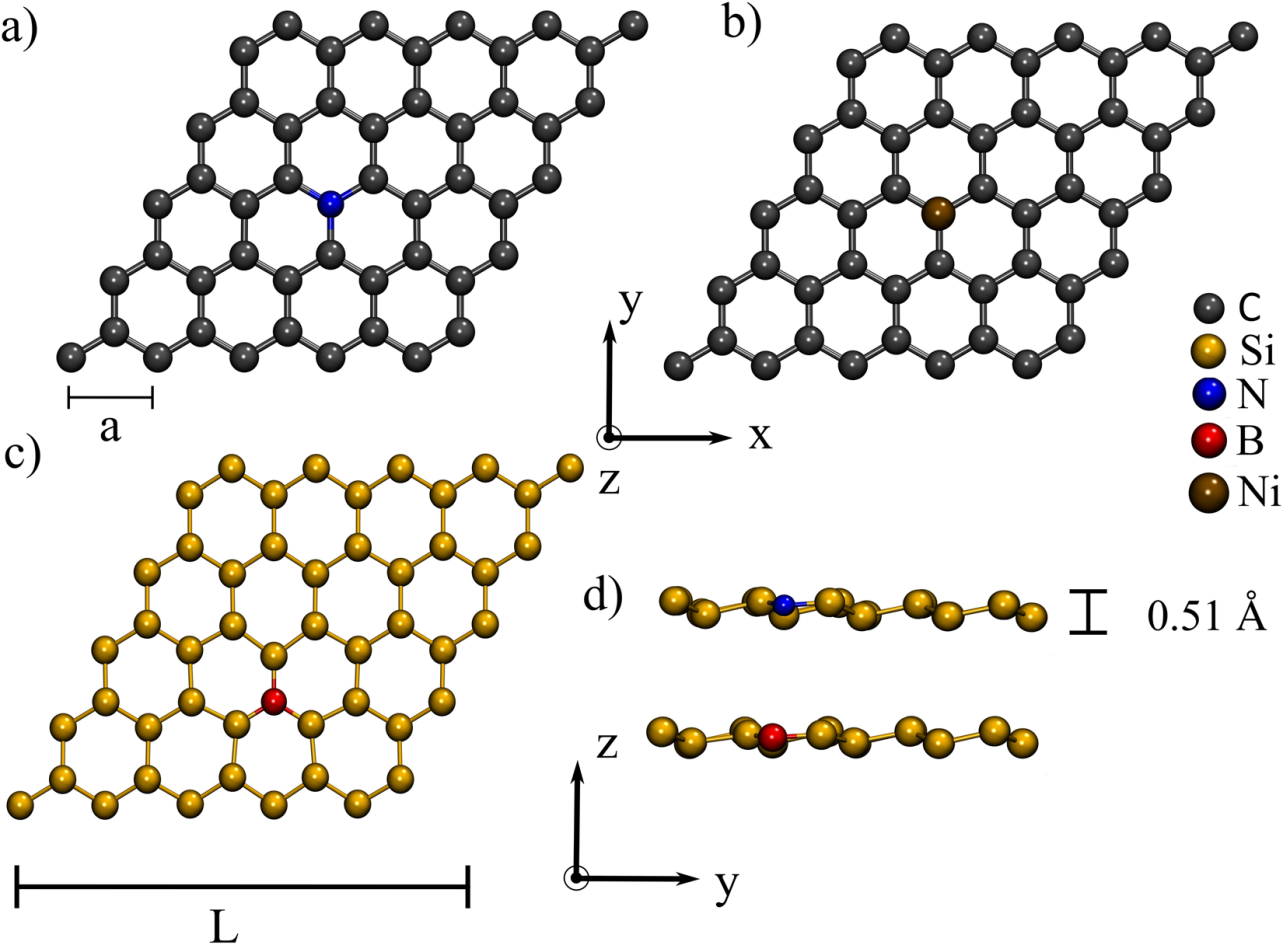
Structures used in the simulations. (**a**) graphene with substitutional nitrogen and boron (not shown) impurities. (**b**) Top-adsorbed Ni atom on graphene. (**c**) Top and (**d**) side views of silicene with nitrogen, or boron substitutional impurities.

The goal of our formulation is to determine energy variation quantities associated to a host material being perturbed by the presence of an impurity. This is done via an analytic approach that gives the energetics of the perturbed system in a transparent functional form. For the sake of illustration, the method will be presented for the case of a graphene sheet (host) doped by a single substitutional atom [cf. Fig. 1(a,b)] but it can be generalized for numerous other hosts that display similar characteristics.

We start off with a generic Hamiltonian $\hat{H}$ containing a perturbation. More specifically, $\hat{H}={\hat{H}}_{0}+\hat{V}$, where

1 $${\hat{H}}_{0}=\sum _{\langle i,j\rangle }|i\rangle {t}_{i,j}\langle j|$$

corresponds to a tight-binding Hamiltonian of the pristine host written in the basis formed by single atomic orbitals |*j*〉 centered at sites identified by the integer *j*. The perturbation potential $\hat{V}=|0\rangle \lambda \langle 0|$ represents a mere shift (by *λ*) in the on-site potential at the impurity site (*j* = 0). The matrix elements *t*_*i*,*j*_ = *t* when *i* and *j* label nearest-neighbour sites but vanish otherwise. As we shall see, the actual value of *t* is arbitrary and immaterial since we are primarily interested in establishing a functional form for the energy variation due to the presence of impurities. The simplicity of this single-orbital picture is in no way a limiting factor since it can be easily generalized to a multi-orbital representation which may also account for further nearest-neighbour couplings if necessary. Likewise, the premise that the introduction of a substitutional impurity does not impact the on-site energies of its neighbours is a consequence of the screening and can be easily relaxed without any qualitative change to our results and conclusions. Attention is drawn to the fact that this Hamiltonian is written in real space and describes a single impurity embedded in an ideally infinite host. This means that $\hat{H}$ still does not carry any of the aforementioned supercell ingredients we eventually want to capture.

The energy cost Δ${\mathcal E}$_1_ associated with the alteration of the host material by a single substitutional impurity can be quantified using

2 $${\rm{\Delta }}{ {\mathcal E} }_{1}=\sum _{\ell }{\int }_{-\infty }^{+\infty }dE\,f(E)\,E\,{\rm{\Delta }}{\rho }_{\ell }(E)\,,$$

where *E* is the energy, *f*(*E*) is the Fermi-Dirac distribution function, and ${\rm{\Delta }}{\rho }_{\ell }$ is the change in the local density of states at site $\ell$. The subscript 1 refers to the introduction of a single impurity onto the host. Δ${\mathcal E}$_1_ is associated with the energy-band contribution to the total energy variation and is present in quantities like the binding energy and formation energy, for instance. Note that the expression above contains a sum over all sites $\ell$ in the host-dopant system. One can benefit from useful mathematical sum rules to rewrite equation (2) in a far more convenient form by working with electronic propagators [or single-particle Green functions (GF)] associated with the Hamiltonian of the pristine host, $\hat{{\mathscr{G}}}={(E-{\hat{H}}_{0})}^{-1}$. The latter can be projected onto any site of the host system. Equation (2) can then be written as^16^

3 $${\rm{\Delta }}{ {\mathcal E} }_{1}=\frac{1}{\pi }{\int }_{-\infty }^{+\infty }dE\,f(E){\rm{Im}}\,\mathrm{ln}\,\mathrm{[1}-{{\mathscr{G}}}_{\mathrm{0,0}}\,\lambda ],$$

with ${\mathscr{G}}$_0,0_ being the GF of the pristine host projected onto the impurity site. Although ${\rm{\Delta }}{\rho }_{\ell }$ obviously depends on $\ell$, the quantity Δ${\mathcal E}$_1_ is not position dependent. Indeed, equation (3) involves only the diagonal matrix element ${\mathscr{G}}$_0,0_ which is identical to any other diagonal element ${{\mathscr{G}}}_{\ell ,\ell }$ and therefore carries no dependence on position.

Following the same line of thought, we compute the energy cost associated with the introduction of a second impurity onto the host. In the case of a pair of identical impurities, the impurity potential becomes $\hat{V}=\mathrm{|0}\rangle \lambda \langle \mathrm{0|}+|\ell \rangle \lambda \langle \ell |$, where one impurity is at site 0 and the other at site $\ell$. The energy cost in this case is given by^15,16^

4 $${\rm{\Delta }}{ {\mathcal E} }_{2}=2{\rm{\Delta }}{ {\mathcal E} }_{1}+C({D}_{\mathrm{0,}\ell }),$$

where the subscript 2 now refers to a pair of impurities and ${D}_{\mathrm{0,}\ell }$ is the distance between them. Notice that Δ${\mathcal E}$_2_ is not simply twice the energy cost of a single impurity but differs by a correction term given by

5 $$C({D}_{\mathrm{0,}\ell })={\int }_{-\infty }^{+\infty }dE\,f(E){\rm{Im}}\,\mathrm{ln}[1-\frac{{\lambda }^{2}\,{{\mathscr{G}}}_{\mathrm{0,}\ell }{{\mathscr{G}}}_{\ell \mathrm{,0}}}{{\mathrm{(1}-\lambda {{\mathscr{G}}}_{\mathrm{0,0}})}^{2}}],$$

with ${{\mathscr{G}}}_{\ell \mathrm{,0}}$ being the off-diagonal GF between the impurity sites. One can keep writing recursively an expression for Δ${\mathcal E}$ as a multiple of Δ${\mathcal E}$_1_ plus pairwise corrections and generalize it to the case in which the host contains *N* impurities

6 $${\rm{\Delta }}{ {\mathcal E} }_{N}=N\,{\rm{\Delta }}{ {\mathcal E} }_{1}+\sum _{\ell ,m}C({D}_{\ell ,m})\,,$$

where the sum now runs over all possible impurity pairs located at sites $\ell$ and *m* and separated by ${D}_{\ell ,m}$. Note that contributions beyond the pairwise interaction terms contained in $C({D}_{\ell ,m})$ have been neglected. This simplification has allowed us to show that the function *C*(*x*) is in general oscillatory and obeys the following functional form *C*(*x*) ∝ cos(*Qx*)/*x*^*α*^, where *Q* and *α* are constants that depend on the geometry of the host’s Fermi surface and on the system dimensionality, respectively^15,17^.

As a result of the oscillatory behaviour of *C*(*x*), one can conjecture that the sum in equation (6) will always average out. Surprisingly, however, when the distance *x* matches any possible pair-distance ${D}_{\ell ,m}$ for sites $\ell$ and *m* on the same sublattice in graphene and other 2D materials, the product $Q{D}_{\ell ,m}$ becomes an integer multiple of 2*π*, which automatically suppresses the oscillatory character of the function *C*(*x*). Therefore, the energetics of graphene with *N* impurities becomes

7 $$\frac{{\rm{\Delta }}{ {\mathcal E} }_{N}}{N}={\rm{\Delta }}{ {\mathcal E} }_{1}+\frac{1}{N}\sum _{\ell ,m}\frac{\gamma }{{({D}_{\ell ,m})}^{\alpha }},$$

where Δ${\mathcal E}$_*N*_/*N* is the energy-cost per impurity and *γ* is a constant. Even though the individual terms in the summation may be substantially smaller than Δ${\mathcal E}$_1_, they add up to a finite quantity when the oscillation of *C*(*x*) is suppressed. In this case, the interaction between impurities can certainly impact on the overall energy balance of the system, leading to Δ${\mathcal E}$_*N*_/*N* ≠ Δ${\mathcal E}$_1_.

One striking consequence of the absence of oscillations in *C*(*x*) is what happens when impurities are orderly distributed. Consider for example a graphene sheet divided into identical diamond-shaped cells of length *L*, all of which containing one substitutional impurity as shown in Fig. 1. The distance between any pair of impurities will be obviously proportional to the size of the unit cells. Therefore, the *L*−dependence of Δ${\mathcal E}$_*N*_/*N* can be easily extracted by simply factoring 1/*L*^*α*^ out of each term in the sum of equation (7). The energy balance equation then becomes

8 $$\frac{{\rm{\Delta }}{ {\mathcal E} }_{N}}{N}={\rm{\Delta }}{ {\mathcal E} }_{1}+\frac{\beta }{{L}^{\alpha }},$$

where *β* is a constant.

While other interactions may play a role in the total energy calculations, they are not as long-ranged as the interaction captured by *C*(*x*) which turns equation (8) into the key result of this manuscript, as explained below. When calculating total energy variations of doped systems within the supercell representation, the quantity that is usually obtained is the energy change per unit cell Δ${\mathcal E}$_*N*_/*N*. This value is expected to coincide with the real quantity of interest, namely Δ${\mathcal E}$_1_, because the last term in the r.h.s of equation (8) is likely to vanish in the limit of sufficiently large values of *L*. This is indeed the case for the vast majority of materials because of the oscillatory behaviour of *C*(*x*). However, this is not so in the case of some 2D materials for which the oscillatory character of *C*(*x*) is suppressed and, consequently, Δ${\mathcal E}$_*N*_/*N* alone is not sufficient to give direct information about Δ${\mathcal E}$_1_. Far from invalidating the supercell methodology, we argue that this feature can be used to accelerate the convergence of such calculations, thereby enhancing its accuracy. By making use of the functional form shown in equation (8), a plot of Δ${\mathcal E}$_*N*_/*N* as a function of *L*^−*α*^ should generate a distinctive straight line with the slope described by *β* and with the intercept given by Δ${\mathcal E}$_1_ in the limit *L* → ∞. To test this, we carried out DFT calculations for different two-dimensional materials doped with a few different impurities. More specifically, graphene and silicene were the 2D materials considered and B, N (substitutional) and Ni (top-adsorbed) atoms were the chosen dopants. We note that, while the lowest energy configuration for Ni on graphene is the hollow hexagonal site, it does not break sublattice symmetry which, as we shall see, turns out to be an essential ingredient for the extrapolation method to work. Thus for the purposes of this work, we consider this metastable structure to demonstrate that our technique is also effective with adsorbed impurities that break sublattice symmetries.

Figure 1 shows the different structures considered in our calculations. In all cases the length of the cell *L* is varied from 4 to 9 or 10 lattice parameters *a*. The electronic structure simulations were conducted with *ab initio* DFT as implemented in SIESTA^5^ with the Generalized Gradient Approximation^18^ (PBE-GGA) for the exchange-correlation functional. The valence electrons are described by a double-*ζ* polarized (DZP) basis set, and norm-conserving pseudopotentials^19^ were used. The cutoff for the real space grid was set at 400, 800, or 1000 Ry, and a k-grid of up to 16 × 16 × 1 was used depending on the size of the unit cell. In order to prevent spurious interaction between image cells, a vacuum region of 30 Å in the transverse direction was introduced. All structures were fully relaxed using a conjugate gradient algorithm with residual forces in each component of every atom smaller than 0.01 eV/Å. For the specific case of adsorbed Ni, graphene was relaxed in its pristine form first and then the Ni atom was placed on it. The Ni was allowed to relax transversely but not the graphene sheet in order to reduce noise in the results.

To check if *ab initio* methods can reproduce the energetic behaviour of equation (8), we consider two different quantities depending on the system being studied; in the case of substitutional impurities, the quantity of interest is the formation energy,

9 $${E}_{{\rm{f}}}(L)={E}_{{\rm{def}}}(L)-{E}_{{\rm{pris}}}(L)+\sum _{i}{n}_{i}{\mu }_{i},$$

where *E*_pris_(*L*) corresponds to the total energy of a pristine 2D host with supercell length *L*, *E*_def_(*L*) is the energy of its counterpart containing the impurity, ${n}_{i}\in {\mathbb{N}}$ is the number of atoms of species *i* removed (${n}_{i} > 0$) or added (*n*_*i*_ < 0) to the system, and finally *μ*_*i*_ is the chemical potential for the corresponding species. The chemical potentials of the N and B impurities were taken considering as reference molecules N_2_ and diborane (B_2_H_6_), respectively. For an adsorbed impurity, we consider another energy quantity, namely the binding energy,

10 $${E}_{{\rm{b}}}(L)={E}_{{\rm{H}}+{\rm{A}}}(L)-{E}_{{\rm{H}}}(L)-{E}_{{\rm{A}}}(L),$$

where *E*_H+A_ corresponds to the total energy of the host with the adatom, and *E*_H_ (*E*_A_) is the total energy of the isolated host (adatom). We note that the functional form of both measures (*E*_f_ and *E*_b_) is the same. Ultimately the choice of chemical potential is somewhat arbitrary and will only introduce a rigid shift on *E*_f_ as it is not dependent on the size of the system. The same is true for the energy of the isolated adatom. Their choice is significant for a quantitative description of the formation/binding energy, but will not interfere in the behaviour of the curve as a function of *L*.

## Results and Discussion

**Figure 2:**
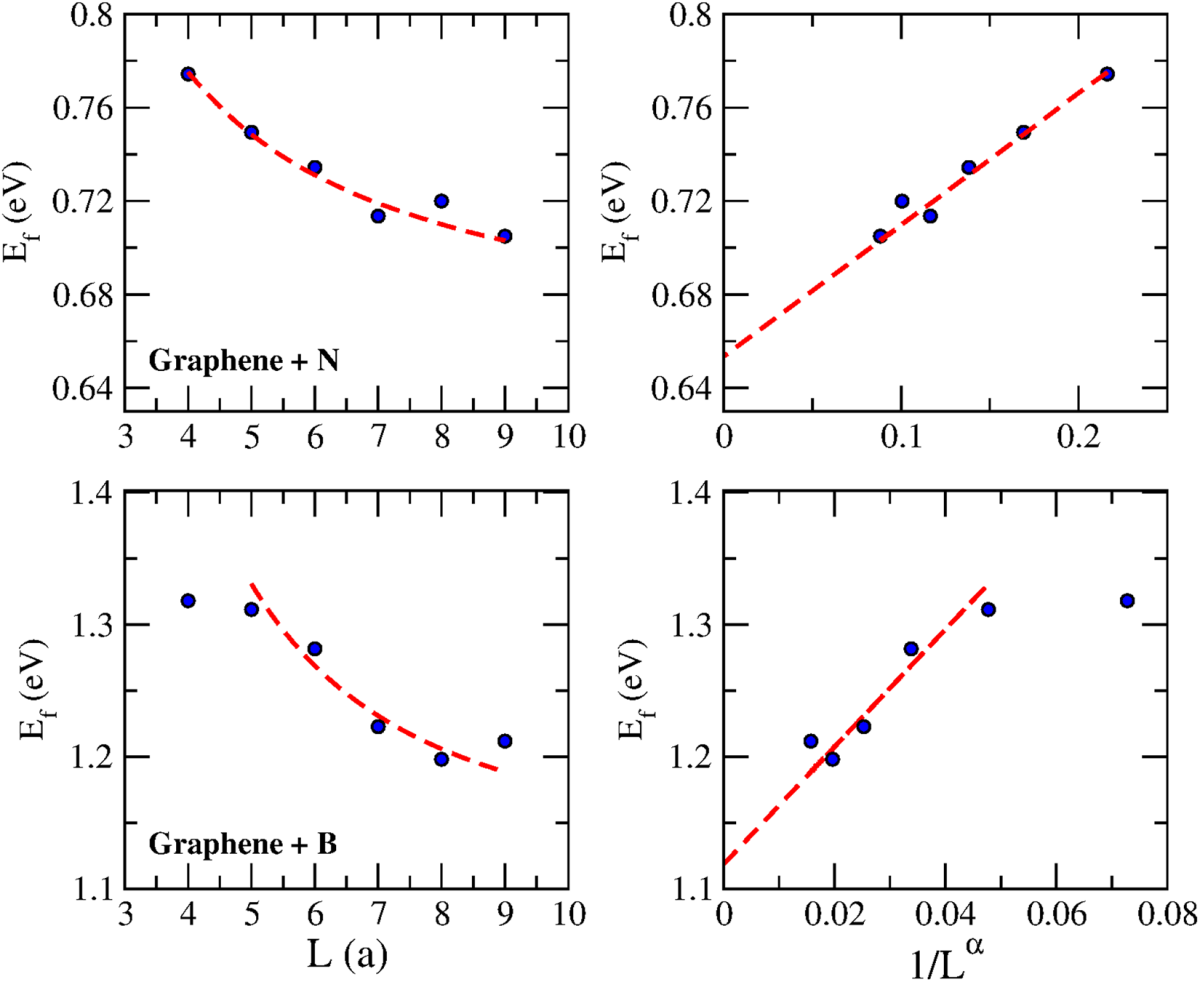
Formation energy (*E*_f_) for graphene doped with substitutional impurities obtained via DFT. Top (Bottom) panels are for N (B) impurities. Left panels display the formation energy *E*_f_ as a function of the unit cell size *L* (in units of the graphene lattice parameter, a). Right panels plot the same results as a function of 1/*L*^*α*^. The exponents are *α* = 1.1 for the case of N and *α* = 1.89 for B. The intercepts of the dashed lines on the right panels are given by Δ${\mathcal E}$_1_ = 0.65 eV and Δ${\mathcal E}$_1_ = 1.12 eV for N and B, respectively.

The first set of results can be seen in Fig. 2 showing the formation energy of graphene doped with substitutional atoms of N (top panels) and B (bottom panels). The left panels display how the DFT-evaluated *E*_f_ changes with the size of the unit cell. Note that in particular for the case of the N-doped system, convergence is rather slow and not reached for *L* < 10*a*, even though there is no direct coupling between images of the impurity atoms for $L > 5a$. Such a slow convergence rate implies that considerably larger unit cells are necessary to achieve reliable values of *E*_f_. Making use of the predicted functional form shown in equation (8), straight lines are found when *E*_f_ is plotted as a function of *L*^−*α*^. By simple extrapolation, the intercept of the linear function provides the exact value of interest, i.e., Δ${\mathcal E}$_1_. Note how different the extrapolated results are from those obtained through a direct calculation with *L* = 9*a*. Such a large discrepancy indicates that this is undoubtedly a very efficient way of reaching convergence without the need for large unit cell calculations.

Our finite-size formulation is by no means exclusive of graphene. To avail of this extrapolation method, certain ingredients are necessary. Obviously, one needs the *L*-dependence of the formation energy as per equation (8). This is a consequence of the lack of oscillations in the function *C*(*x*) describing the interaction between impurities which, in turn, is linked with the existence of Dirac points. In other words, systems whose electronic structure possesses Dirac points^20–22^ or for which the corresponding Fermi surface lies on the edges of the Brillouin zone are most likely to display these features. Furthermore, another essential ingredient is that the perturbing potential associated with the impurity must impact sublattices asymmetrically. This latter requirement is obviously met by the introduction of substitutional impurities onto the host.

**Figure 3:**
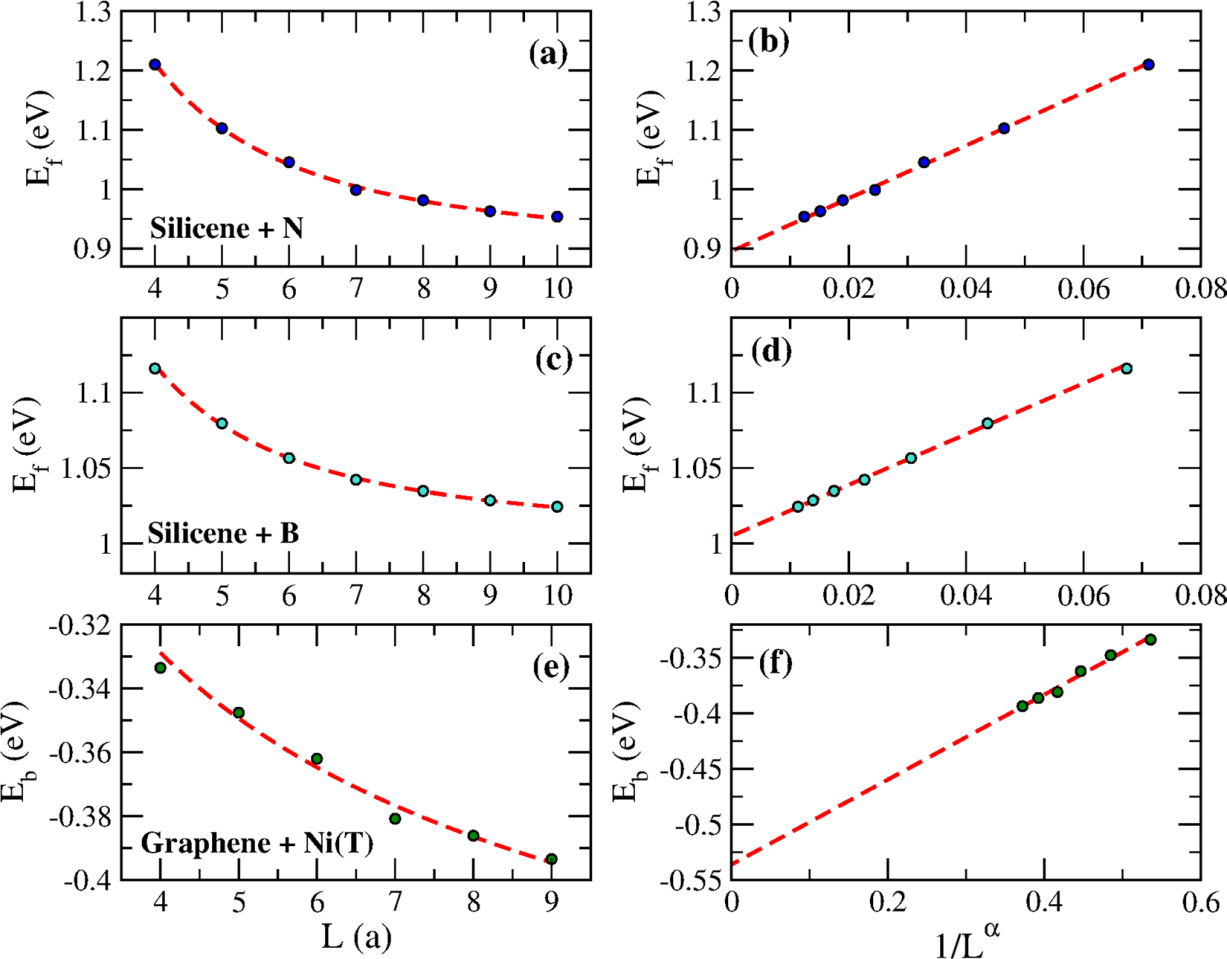
Top and middle panels show the formation energy (*E*_f_) for silicene doped with substitutional impurities obtained via DFT. (a,b) panels are for N dopant whereas (c,d) panels are for B dopant. Left panels display the formation energy *E*_f_ as a function of the unit cell size *L* (in units of the host lattice parameter, *a*). Right panels plot the same results as a function of 1/*L*^*α*^. The exponents are *α* = 1.91 for the case of N and *α* = 1.95 for B. The intercepts of the dashed lines on the right panels are given by Δ${\mathcal E}$_1_ = 0.89 eV and Δ${\mathcal E}$_1_ = 1.01 eV for N and B, respectively. The bottom (e,f) panels show the binding energy (*E*_b_) of a Ni impurity top-adsorbed (T) on graphene obtained through DFT. The exponent of *α* = 0.45 and an intercept of Δ${\mathcal E}$_1_ = −0.54 eV were found in this case.

To illustrate this point we demonstrate that the extrapolation technique works equally well with systems containing both of these necessary ingredients, namely the Dirac-point and the sublattice-asymmetry requirements. Silicene is one ideal candidate since it has Dirac points. The top and middle panels of Fig. 3 show DFT results for the formation energy of silicene doped with substitutional atoms of N and B, respectively. Once again, a slow convergence rate is found on the left panels as the unit cell size *L* is increased. However, very distinctive straight lines are visible when *E*_f_ is plotted on the right panels as a function of 1/*L*^*α*^, from which we can easily extract the intercept values through extrapolation. Similarly, the discrepancy between the extrapolated values and those obtained with *L* ≈ 10*a* is significant and ranges at approximately 25 meV.

Substitutional impurities are not the only type giving rise to sublattice-asymmetric perturbing potentials. Adatoms are known to adsorb to 2D materials at different locations of their atomic structure. Top-adsorbed impurities are those attached more strongly to one of the host atoms and should meet the requirement of sublattice asymmetry in the perturbing potential. We put this requirement to the test by calculating the binding energy of Ni impurities that were top-adsorbed to graphene (cf. Fig. 1). The bottom panels of Fig. 3 display the DFT-calculated results. Again, we note the straight line as *E*_b_ is plotted as a function of the rescaled quantity 1/*L*^*α*^.

Simple dimensionality arguments can be used to justify that the value of the exponent should be *α* = 2^23^. However, the values of *α* used to transform the energetic curves into straight lines are not the same and differ depending on the host, on the impurity and on the bonding geometry, as shown in Figs 2 and 3. The variability of *α* is a consequence of the simplifying assumptions made in the derivation of equation (8). For a start, we assumed that efficient screening makes the perturbing potential *V* active only locally. This assumption is partially valid since DFT calculations involve real-space matrix elements that go beyond the immediate vicinity of the impurity. With this assumption relaxed, the potential *V* is not as localized and involves sites from both sublattices of the hexagonal structure. Contributions to the formation/binding energy coming from distinct sublattices are known to have opposite signs^16^ and this will inevitably reduce the strength of the interaction. As a result, the functional form of equation (8) still holds but with an exponent that is not necessarily determined only by the system dimensionality. Another simplifying assumption was to consider the system Fermi level precisely at the Dirac point. Any amount of charge transfer between host and dopant is likely to shift the Fermi level of the doped system away from the Dirac point. The argument that preceded equation (7) and which explained the suppression of the oscillatory character of the function *C*(*x*) is no longer applicable when the Fermi level shifts from the Dirac point. Nevertheless, if the charge transfer and consequently the Fermi-level shift are sufficiently small, the infinite series that appears in the r.h.s. of equation (7) becomes an alternating series that does not vanish but converges to a finite contribution. Once again, equation (8) maintains its form with the Dirac-point assumption relaxed but with a to-be-determined exponent.

The exact value of *α* is difficult to find from first principles but can be obtained through a fitting procedure in which a power law function with three floating parameters are used. These parameters are the quantities appearing on the r.h.s of equation (8), i.e., Δ${\mathcal E}$_1_, *β* and *α*. Rather than carrying out a blind search for the exponent that transforms the DFT-evaluated *E*_f_(1/*L*^*α*^) into a linear function, we constrain the fitting scheme to search for *α* in close proximity to the dimensionality-defined value of *α* = 2. This was the procedure carried out in every single case shown in Figs 2 and 3, all of which display excellent results. A formal derivation of the exponent *α* and how it depends on the charge transfer between host and impurity is a challenging task that deserves attention of the community working in this field. Once established, it will provide accurate values of formation and binding energies from computations involving only very small unit cell sizes. Meanwhile, even without full knowledge of how *α* scales with the charge transfer, calculations can be immensely simplified by the extrapolation scheme presented here.

In summary, we have derived a useful expression for the change in total energy due to the formation of an array of periodically spaced impurities in 2D materials. This expression captures how quantities like formation energy and binding energy scale with the size of the unit cell, which is so commonly used in supercell implementations of *ab-initio* calculations. Moreover, we demonstrate that these quantities may display very slow convergence rates when they are calculated through the usual procedure of increasing the size of unit cells for the specific case of systems that present a commensurability effect between the impurity-induced Friedel oscillations and the lattice. When that happens, prohibitively large unit cells may be needed to reach desirable accuracies. Rather than invalidating the methodology that is so prevalent in electronic-structure calculations of 2D materials, we argue that we can make the most of that slow convergence rate to introduce an extrapolation scheme that provides results with accuracy levels that would otherwise require enormously large unit cells. Furthermore, our expressions were derived with doped graphene sheets in mind but our approach is by no means exclusive of that material and has also been shown to work for other 2D structures with different types of dopants, namely substitutional and adsorbed impurities. Finally, although our conclusions were based on the DFT results used throughout this paper, any other computational tool implemented within the supercell representation is bound to display exactly the same behaviour and can be optimized through the proposed extrapolation scheme.
